# Postpolypectomy Syndrome Without the Use of Hot Snare

**DOI:** 10.7759/cureus.79010

**Published:** 2025-02-14

**Authors:** Ryan D Plunkett, Neel Matiwala, Matthew Schroeder, Amna Qureshi, Michael Presti

**Affiliations:** 1 Internal Medicine, Saint Louis University School of Medicine, St. Louis, USA; 2 Gastroenterology and Hepatology, Washington University School of Medicine in St. Louis, St. Louis, USA; 3 Pathology, John Cochran Division, Veterans Affairs Medical Center, St. Louis, USA; 4 Gastroenterology and Hepatology, St. Louis Veterans Affairs Medical Center, St. Louis, USA

**Keywords:** clinical case report, cold biopsy forceps polypectomy, cold snare polypectomy, hot snare polypectomy, postpolypectomy syndrome (pps)

## Abstract

The mechanism of postpolypectomy syndrome (PPS) has been largely attributed to transmural injury related to electrocoagulation. This syndrome classically presents with fever, leukocytosis, localized peritoneal inflammation, and abdominal pain following colonoscopy with polypectomy, resolving with conservative management. However, this case describes a patient who presented with these symptoms after undergoing resection of 11 polyps via cold snare and biopsy forceps. Histology did not show deep colonic injury from the polypectomy, and no electrocoagulation was used, making this case inconsistent with the accepted explanation of postpolypectomy syndrome.

## Introduction

Postpolypectomy syndrome (PPS, also known as postpolypectomy electrocoagulation syndrome) presents with fever, localized abdominal pain, regional peritoneal signs, and leukocytosis typically within hours to days post-colonoscopy with polypectomy. Classically, PPS is associated with hot snare polypectomy (HSP) as the electrocoagulation in HSP serves as the postulated insult resulting in a transmural burn. The reported incidence of this rare complication varies widely from 0.07% to 9.7%, with higher rates seen with polyps larger than 10 mm [1,2].

The ability of clinicians to recognize PPS is crucial as its presenting symptoms often include peritoneal signs, which can lead to unnecessary operative interventions if misdiagnosed. Abdominal computed tomography (CT) is an imperative tool for PPS evaluation as it can effectively rule out visceral perforation. Standard PPS management is conservative, that is, bowel rest, intravenous hydration, and empiric antibiotic coverage for colonic flora with the advancement of diet as symptoms improve. Current guidelines do acknowledge the associated risks of HSP, such as bleeding, perforation, and PPS. Therefore, European endoscopic guidelines recommend cold snare polypectomy (CSP) for diminutive polyps of ≤5 mm and sessile polyps of 6-9 mm [1]. However, despite this known connection between HSP and PPS, we present an unusual case of postpolypectomy syndrome following the removal of 11 polyps with cold snare and biopsy forceps.

## Case presentation

A 70-year-old man with a past medical history of nicotine dependence, hypothyroidism, hypertension, type 2 diabetes mellitus, chronic obstructive pulmonary disease, coronary artery disease, and gastroesophageal reflux disease presented to the emergency department with acute lower abdominal pain. The patient described 10/10, intermittent, non-radiating pain localized to the right lower quadrant. The pain started hours after a colonoscopy the day prior. He was able to tolerate oral intake and denied nausea, vomiting, melena, bright red blood per rectum, or any history of similar symptoms. The patient appeared comfortable on examination, only endorsing tenderness to palpation in the right lower quadrant with no overt peritoneal signs.

**Figure 1:**
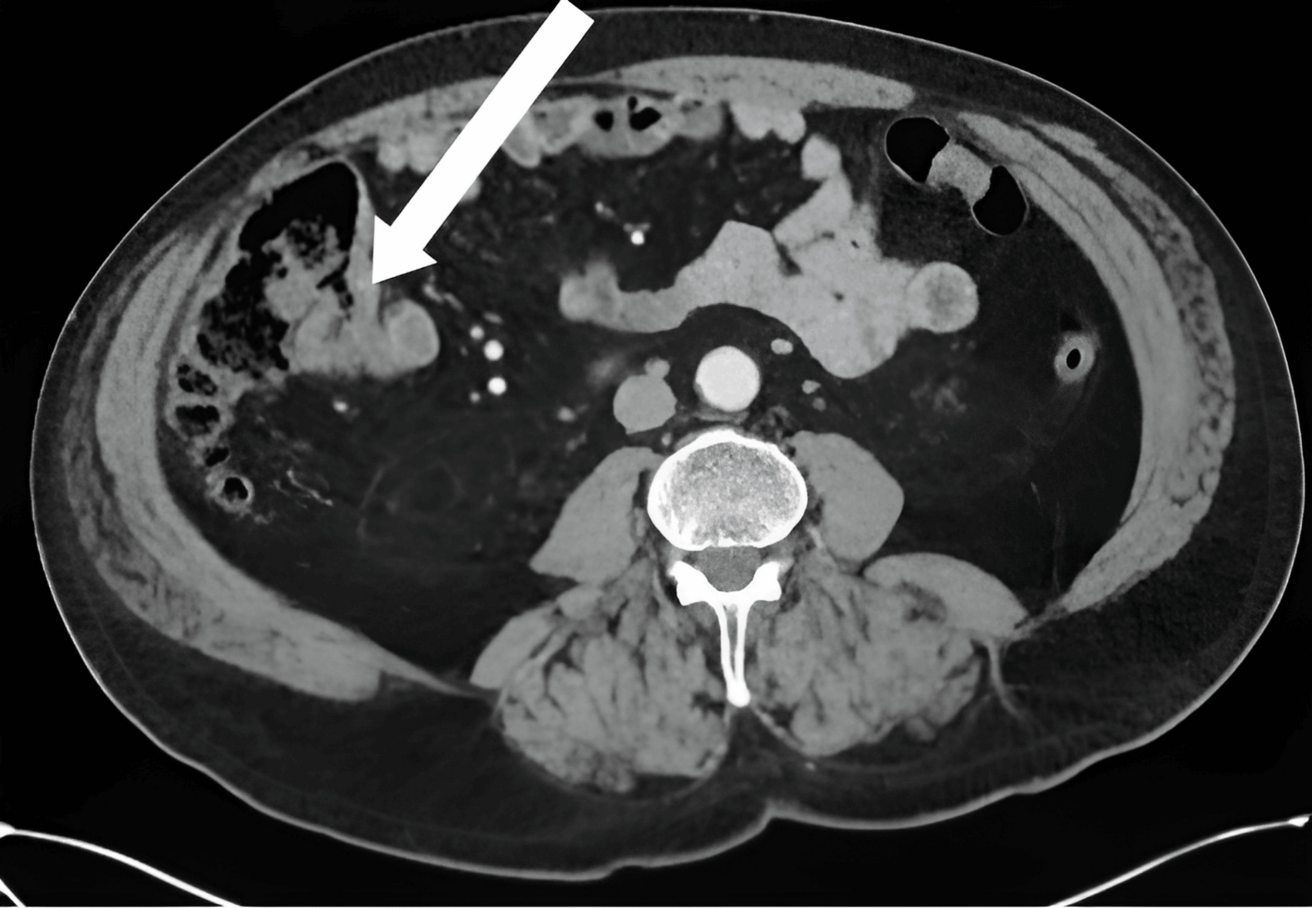
Mild focal-appearing nonspecific wall thickening at the cecal base (arrow).

Vital signs were stable, he remained afebrile, and laboratory results were only notable for a mildly elevated white blood cell count. CT of the abdomen and pelvis showed nonspecific focal thickening at the cecal base with possible adjacent fat stranding with concern for postpolypectomy syndrome (Figure 1). No free air or loculated collections were seen.

The colonoscopy was a five-year follow-up after the removal of hyperplastic colon polyps without high-risk adenomas. Per the detailed procedure report, 11 2-6 mm polyps were removed via a combination of cold snare and biopsy forceps without complication, without the use of hot snare, as demonstrated in Table 1.

**Table 1:** Details of the number, size, location, pathology, and method of polypectomy from the colonoscopy.

Polyps Removed	Polyp Size (mm)	Polyp Location	Polyp Pathology	Removal Method
1	4	Cecum	Tubular Adenoma	Cold Biopsy Forceps
3	2-3	Ascending Colon	Tubular Adenoma	Cold Biopsy Forceps
2	5-6	Transverse Colon	Tubular Adenoma	Cold Snare
1	3	Descending Colon	Hyperplastic Polyp	Cold Biopsy Forceps
3	6	Sigmoid Colon/Rectum	Hyperplastic Polyp	Cold Biopsy Forceps

Although no electrocautery was used, the constellation of leukocytosis, abdominal pain, and mild cecal thickening correlating with a polypectomy site prompted concern for postpolypectomy syndrome. The patient was started on a clear liquid diet, isotonic fluids, ceftriaxone, and metronidazole with a gastroenterology consultation. The following day, the leukocytosis resolved. The patient tolerated a clear liquid diet and was passing flatulence, and his abdominal pain had significantly improved. He was discharged on a five-day course of ciprofloxacin and metronidazole. On follow-up with primary care one month later, the patient’s symptoms had completely resolved with no lingering effects.

## Discussion

In this case, PPS was suspected given the patient’s abdominal pain, leukocytosis, and CT findings of localized inflammation and edema. Despite having multiple polyps removed throughout the colon, our patient only developed right-sided tenderness, which is consistent with the diagnosis as PPS preferentially affects the right colon given the region’s thinner wall [1].

**Figure 2:**
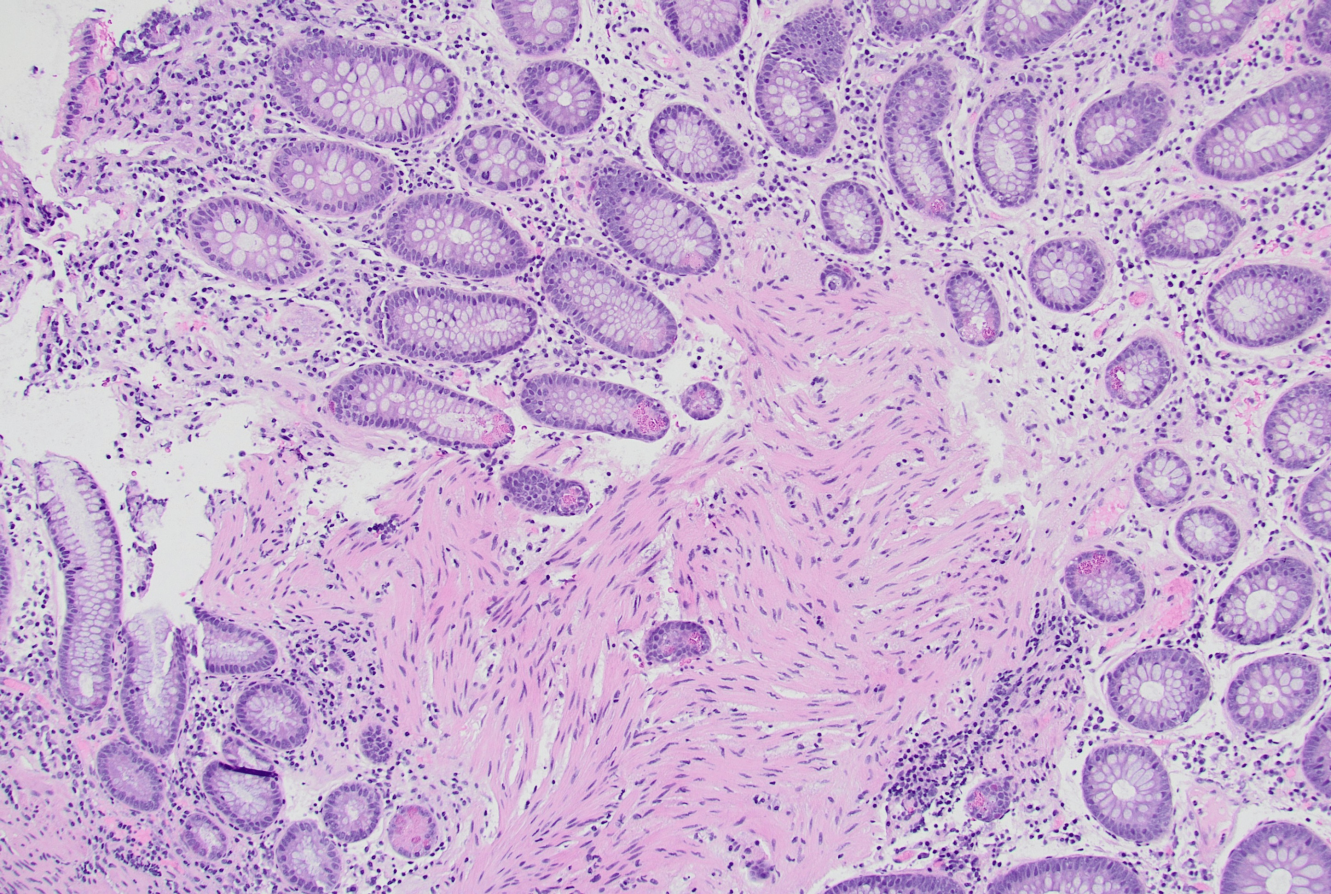
Cecal biopsy at 10× magnification showing colonic mucosa with mild chronic inflammation and bands of smooth muscle representing muscularis mucosa.

Prior studies have undoubtedly shown higher rates of PPS following HSP and found mucosal injury to be deeper in HSP than in CSP, correlating with the idea of transmural insult leading to this syndrome [2]. However, when reviewing histology from our case, the deeper injury was not shown, with polypectomy depth only extending to the muscularis mucosa (Figure 2). Therefore, it is worth reviewing other reported instances where PPS has occurred after CSP, which may call the mechanistic understanding of PPS due to deep, transmural burn into question. One case describes a patient with right lower quadrant pain and ascending colonic thickening on CT after cold snare endoscopic mucosal resection (EMR) with submucosal epinephrine injection of a 7 mm sessile serrated lesion [3]. Another report highlights apparent PPS in a patient with familial adenomatous polyposis who underwent CSP of 50 <5 mm sessile rectal polyps [4]. Our case occurred after 11 cold resections, with cold forceps polypectomy of four 2-4 mm lesions near the region of detected inflammation.

Within the literature, multiple PPS risk factors have been described such as hypertension, non-polypoid lesions, pedunculated polyps, serrated polyps, the absence of intraepithelial neoplasia, cecal resections, longer electrocautery duration, higher polypectomy quantity, and the capture of adjacent normal mucosa [2,5]. The consideration of these risk factors has led to the development of additional hypotheses for the cause of PPS. These include inflammatory cytokine reactions from large polypectomies and bacterial translocation through submucosal injection or microbiota interactions with mucosal injuries [2]. Though intriguing, these claims have their limitations as supportive trial data is insufficient.

## Conclusions

While HSP is associated with PPS, multiple cases have now been reported with similar clinical syndromes without HSP. Given these cases and risk factors such as polypectomy quantity and adjacent tissue affected, it may be worth considering if the inherent effects of polypectomy and exposure to mucosal injury can contribute to the pathology of PPS, rather than purely transmural burn.
